# Mismatch Responses in the Awake Rat: Evidence from Epidural Recordings of Auditory Cortical Fields

**DOI:** 10.1371/journal.pone.0063203

**Published:** 2013-04-30

**Authors:** Fabienne Jung, Klaas Enno Stephan, Heiko Backes, Rosalyn Moran, Markus Gramer, Tetsuya Kumagai, Rudolf Graf, Heike Endepols, Marc Tittgemeyer

**Affiliations:** 1 Max-Planck-Institute for Neurological Research, Cologne, Germany; 2 Translational Neuromodeling Unit (TNU), Institute for Biomedical Engineering, University of Zurich and Swiss Federal Institute of Technology (ETH) Zurich, Zurich, Switzerland; 3 Laboratory for Social and Neural Systems Research (SNS), University of Zurich, Zurich, Switzerland; 4 Wellcome Trust Centre for Neuroimaging, University College London, London, United Kingdom; 5 Department of Neurosurgery, Osaka University Graduate School of Medicine, Osaka, Japan; University of Salamanca- Institute for Neuroscience of Castille and Leon and Medical School, Spain

## Abstract

Detecting sudden environmental changes is crucial for the survival of humans and animals. In the human auditory system the mismatch negativity (MMN), a component of auditory evoked potentials (AEPs), reflects the violation of predictable stimulus regularities, established by the previous auditory sequence. Given the considerable potentiality of the MMN for clinical applications, establishing valid animal models that allow for detailed investigation of its neurophysiological mechanisms is important. Rodent studies, so far almost exclusively under anesthesia, have not provided decisive evidence whether an MMN analogue exists in rats. This may be due to several factors, including the effect of anesthesia. We therefore used epidural recordings in awake black hooded rats, from two auditory cortical areas in both hemispheres, and with bandpass filtered noise stimuli that were optimized in frequency and duration for eliciting MMN in rats. Using a classical oddball paradigm with frequency deviants, we detected mismatch responses at all four electrodes in primary and secondary auditory cortex, with morphological and functional properties similar to those known in humans, i.e., large amplitude biphasic differences that increased in amplitude with decreasing deviant probability. These mismatch responses significantly diminished in a control condition that removed the predictive context while controlling for presentation rate of the deviants. While our present study does not allow for disambiguating precisely the relative contribution of adaptation and prediction error processing to the observed mismatch responses, it demonstrates that MMN-like potentials can be obtained in awake and unrestrained rats.

## Introduction

In a volatile environment, fast and automatic detection of sudden changes is crucial for the survival of any animal. In the auditory domain, such a detection mechanism is reflected by the mismatch negativity (MMN). The MMN has classically been defined as a component of auditory evoked potentials (AEPs) that is elicited by unexpected stimuli (“oddballs” or “deviants”) occurring within a stream of homogeneous and predictable sounds (“standards”) [1]. Standard and deviant stimuli can differ in various dimensions like, for example, carrier frequency, intensity or duration [2]. Critically, the MMN is not only elicited by deviations from regular stimulus trains, but by any violation of established expectancies or predictions, including abstract rules (for review, see Garrido et al. [3]). Today, it is therefore often interpreted as a prediction error signal that is generated when an incoming acoustic stimulus violates a prediction based on the past auditory sequence. This has also been referred to as the “model adjustment” theory which views the MMN as reflecting the adjustment of a probabilistic model which the brain constructs and continuously updates to predict future auditory inputs [4]–[6]. A second main hypothesis of MMN explains its generation by a local neurophysiological effect, i.e., stimulus-specific adaptation (SSA) of neurons in primary auditory cortex [7]–[10]. Response amplitudes seem to be reduced for repeated stimuli but also for stimuli that are only similar to the one previously presented. Different or new stimuli, however, are able to restore the initial response amplitude. Moreover, this process is sensitive to the presentation rate of acoustic stimuli. Overall, from the available experimental evidence and recent modeling studies, it appears that both proposed mechanisms play a role in MMN generation [3], [11]. These competing accounts have recently been combined in a unified explanation of MMN. This is a predictive coding framework in which the MMN reflects a prediction error dependent updating of a hierarchical model that infers the causes of sensory stimuli and predicts future inputs [12], [13]. In this theory of MMN generation, model adjustment corresponds to prediction error dependent synaptic plasticity of connections between hierarchically related regions (such as primary and secondary auditory cortex), and adaptation serves to balance the postsynaptic sensitivity to top-down predictions and bottom-up stimulus information, respectively. In other words, adaptation-like mechanisms may act locally in the auditory cortex and modulate how ascending fibers transmit prediction error to higher cortical levels and how descending connections provide contextual guidance to lower levels (i.e., transmit predictions). In this view, the MMN represents a failure to predict bottom-up inputs and suppress prediction error [11], [14].

Given that it provides experimental access to mechanisms of probabilistic inference in the brain, the MMN has gained considerable interest in cognitive neuroscience in the past. Perhaps more importantly, however, it shows prominent alterations in numerous brain diseases, including dyslexia [15] and schizophrenia [16], [17]. Given its simplicity, robustness and the existence of formal models for its generation, it has great translational potential for clinical neuroscience. To unlock this potential, we need to understand the neuronal mechanisms of MMN generation in detail; beyond human experiments with EEG, this requires invasive recordings in animal studies and pharmacological perturbations. However, for making appropriate use of animal data, we first need to establish under which conditions MMN responses can be obtained that are comparable to human MMN.

MMN-like responses have been successfully obtained in recordings from non-human primates (macaques, [18], [19]), rabbits [20], cats [21], mice [22] and guinea pigs [23]. In contrast, MMN studies in rats provide somewhat inconsistent results. For example, differences in the polarity and time course of mismatch responses were found across studies, leading to an ongoing controversy whether MMN-like responses exist at all in rats (for a review see Nelken and Ulanovsky [24]). However, this discrepancy across studies may be due to several factors, including differences in recording sites, stimulus properties, experimental design and anesthesia. The latter is a particularly important factor because response properties of auditory neurons can change drastically under anesthesia [25]–[27]. Clearly, it is experimentally much more challenging to record from awake rats which may explain why electrophysiological recordings of responses to oddball stimulation in the absence of anesthesia are rare [28]–[30].

Concerning experimental design, the traditional way to evoke MMN with a frequency mismatch is a “flip-flop” design in which the frequencies of standard and deviant acoustic stimuli are swapped in two consecutive sessions. Here, in order to control for effects of carrier frequency, MMN is defined as the difference between the averages (across sessions) of the standard-evoked and the deviant-evoked potentials. Furthermore, in several rat studies, the MMN is operationalized as the difference between (averaged) deviant responses and responses to a “deviant alone” condition in which deviants are presented without standards to remove the predictive context. Using this design in rats, Ruusuvirta et al. [31] detected mismatch responses under urethane anesthesia. In contrast, Lazar and Metherate [32] did not find MMN responses under similar conditions, while Tikhonravov et al. [33] [34] recorded MMN-like potentials under pentobarbital-sodium anesthesia. The deviant control condition, however, seems to be unnecessarily strict (see for example Nelken and Ulanovsky [24]). Astikainen et al. [35] report mismatch responses in urethane anesthetized rats for frequency and intensity deviants by comparing deviants to standards only. In addition, there is one paper reporting mismatch responses to duration deviants in awake rats [36].

Numerous variations of this classical MMN design have been proposed [37], [38], and several additional control conditions have been suggested in an attempt to clarify the relative contributions of specific mechanisms. One of these controls is the “deviant within many standards” developed by Jacobsen and Schröger [39]. In this condition the overall presentation rate of deviants is the same as in the oddball condition but standards are replaced by a number of acoustic stimuli with different frequencies. Each stimulus is presented with the same probability and in a random manner so that no regularity is formed. Using this condition Astikainen et al. [40] confirmed the existence of mismatch-responses recorded epidurally in urethane anaesthetized rats. Similarly, mismatch responses were also reported by Nakamura et al. [28] with epidurally recorded potentials in awake and anesthetized rats.

All rat studies described above used only a single epidural recording electrode and could therefore not investigate different regions of the auditory cortex separately. This is a limitation because studies in other species (e.g., guinea pigs [23] and cats [41]) have shown that MMN responses are not only generated by primary auditory cortex, but also by secondary auditory areas.

In this study, we make the first attempt to record MMN in awake rats from two auditory areas bilaterally. For this purpose, we set up a system for wireless electrophysiological recordings from the cortical surface of awake and unrestrained rats. We used four epidural electrodes, two in each hemisphere, above the primary auditory cortex and above the posterior auditory field [42] to investigate oddball elicited potentials generated in the primary and secondary auditory cortex. Furthermore, we chose bandpass-filtered noise stimuli comprising frequencies that are perfectly suitable for the rats’ hearing range. In this way, we aimed to overcome methodological problems present in other studies (e.g. [28] see discussion in their paper) with acoustic stimuli located at the lower end of the rats’ hearing range. Using the classical oddball paradigm and the additional control condition described above, we report large differences between deviant- and standard AEP that are present at all four electrodes and share some key morphological and functional characteristics with human MMN. These mismatch responses significantly diminished in a control condition that removed the predictive context while controlling for presentation rate of the deviants and overall duty cycle.

## Materials and Methods

### Subjects and Surgery

Experiments were performed on 16 male black hooded adult rats (Janvier, France) weighing between 260 and 410 g at the day of surgery. Animals were housed with an inverse 12 hours day-night cycle with lights on at 8∶30 pm in a temperature (22±1°C) and humidity (55±5%) controlled room. Prior to surgery the animals were housed pairwise in type 4 cages filled with Lignocel® (hygiene animal bedding) enriched with nestboxes and horizontal tubes for climbing. After surgery, the animals were kept in pairs, but nestboxes and tubes were removed to reduce the risk of tearing off the implanted telemetry sockets. In addition, the cages were equipped with elevated lids after the operation.

In a series of pretests the hearing ability of rats was determined by brainstem audiometry. After we found large differences regarding the hearing thresholds of rats purchased from different breeders (up to 40 dB difference) we chose those rats that exhibited lowest hearing thresholds (38 dB pSPL) from the three groups tested.

All rats were chronically implanted with epidural silverball electrodes under inhalation anesthesia (isoflurane 2–3% mixed with 30% oxygen (O_2_) and 70% nitrous oxide (N_2_O)). Prior to surgery, rats were given an i.p. injection of 5 mg/kg Carprophen (Rimadyl) as analgetic. For placing the electrodes the temporalis muscle was partly removed and a cranial window was opened with a dental drill. Guided by stereotaxic coordinates two electrodes were positioned above the right and two above the left hemisphere. They covered the primary auditory area, A1 (coordinates relative to bregma: 4 mm posterior, 8 mm lateral, 4 mm ventral) and the posterior auditory field, PAF (6 mm posterior, 8 mm lateral, 4 mm ventral), thereby targeting a primary and a non-primary auditory cortex, respectively [42].

A reference electrode was placed 5 mm anterior to bregma at midline over the frontal sinus. The telemetry socket, to which electrodes were soldered, was fixed onto the skull with dental cement. Before terminating anesthesia, rats were given an additional analgetic (0.3 mg/kg Buprenorphine (Temgesic), i.p.). For three days after the surgery, the animals received painkillers (0.3 mg/kg Buprenorphine (Temgesic), i.p.) once a day and 5 mg/kg Carprophen (Rimadyl) twice. Animals were allowed to recover for ten days after surgery and weighed regularly to assure that they were eating normally.

All experimental procedures were approved by the local governmental and veterinary authorities of Cologne (file number 9.93.2.10.35.07.056). All information required according to the ARRIVE guidelines [43] are included.

### Acoustic Stimuli and Paradigm

Acoustic stimuli were presented with Tucker Davis Technologies® (TDT) System 3 and delivered via free-field magnetic speakers (FF1, TDT). Sounds were generated with the program SigGen (TDT) as white noise and bandpass filtered to 7–9 kHz (low frequency stimulus) and 16–18 kHz (high frequency stimulus). We used bandpass filtered noise rather than sine tones since neurons in the auditory cortex adapt rapidly to pure tone stimuli, and we wanted to ensure the largest response amplitude over time. The frequencies chosen match the rats' hearing ability [44].

In an initial series of experiments with four rats, acoustic stimuli of various durations (50 ms to 120 ms in 10 ms steps) were used in an oddball paradigm (deviant probability 0.1). These initial tests guided our stimulus choice for the main study where we chose 100 ms stimulus length with 10 ms rise and fall time. The stimuli were played with SigPlay32 (TDT) using a presentation rate of 2 Hz. The stimuli were calibrated using a microphone (model 7016, ACO Pacific, Belmont, California) and adjusted to 75 dB SPL with a SPL-meter (NL 32, RION Co. Ltd, Tokyo Japan) placed in the middle of the rats' recording cage.

In the oddball paradigm using all animals, we used a classical flip-flop design with four experimental blocks, each block comprising 1000 stimuli. In the first session the low frequency stimulus (7–9 kHz) was used as standard (standard f1) and the high frequency stimulus as deviant sound (16–18 kHz, deviant f2). In the subsequent block, stimuli were swapped so that the high frequency stimulus served as standard (standard f2) and the low frequency stimulus as deviant (deviant f1). For investigating the effect of deviant probability on the detected differences we tested two different deviant probabilities: 0.1 (100 deviant stimuli) and 0.2 (200 deviant stimuli). The order of experimental blocks was counterbalanced across animals and blocks belonging to the same deviant probability were analyzed as one pair.

Differences between standard and deviant potentials can be due to either the rarity of the deviant (i.e., less adaptation or refractoriness) or the violation of predictions based on the previous acoustic sequence. In order to distinguish between these two mechanisms we used the control condition suggested by Jacobsen and Schröger [39] (“deviant in many standards”). The overall presentation rate of deviants is preserved in this paradigm but standards are replaced by stimuli with different carrier frequencies. The term “deviant in many standards” might be misleading because each stimulus is presented with the same probability and in random manner so that no stimulus functions as deviant or standard. Therefore, we use the term “equiprobable control condition” as suggested by Astikainen et al. [40]. We designed one condition to match each deviant probability that was used in the classical oddball paradigm: For the control condition with deviant probability 0.1, we used 10 different band-pass filtered noise stimuli (7–9, 8–10, 9–11, 10–12, 11–13, 12–14, 13–15, 14–16, 15–17, 16–18 kHz), each presented 100 times in random order (equiprobable control 0.1). The second control with deviant probability 0.2 comprised 5 stimuli (7–9, 10–12, 13–15, 16–18, 19–21 kHz) each presented 200 times in random order (equiprobable control 0.2). Recordings during acoustic stimulation with the two control protocols were done in 6 rats (belonging to the group of 16 rats that were used in the main oddball experiments).

### Electrophysiological Recordings

Recordings were performed during the active phase of the rats, i.e., the dark phase. The transmitter (TSE Systems GmbH, Bad Homburg, Germany) that transferred the recorded EEG signal telemetrically (frequency 417 MHz) to an antenna, had to be attached to the implanted socket prior to each experiment. For connecting the transmitter with the socket, rats were anesthetized briefly with isoflurane. The EEG signal was preamplified in the transmitter (1000×) and amplified again in the receiver (10×). The data were automatically bandpass filtered (0.6–60 Hz) within the telemetry system (TSE, Bad Homburg, Germany). The bandpass filter was implemented in the telemetry system and set to a fixed value.

During the recordings, animals were placed in a wire cage (21×35×22 cm) located between the loudspeakers. Speakers were mounted at a height of 10 cm and ±25 cm distant from the middle of the cage. All experiments were performed in a sound attenuated chamber.

### Data Analysis

For acquiring and storing the data we used a Windows computer with the program DasyLab® (Version 9.0, National instruments, Austin, Texas).

Data were initially sampled with 2 kHz and afterwards downsampled to 1 kHz for the offline analysis using MATLAB® (Version 2011b, Mathworks, Natick, Massachusetts). Electrodes that recorded no evoked activity (altogether 4 electrodes) were eliminated from the analysis. Average evoked potentials were calculated for each animal separately for standard stimuli (mean over all standards f1 and f2) and deviant stimuli (mean over all deviants f1 and f2). Baseline-correction of evoked potentials was done by subtracting the average value of the 100 ms baseline of each potential. Mismatch responses were calculated as deviant minus standard-evoked potential.

For analysis of the equiprobable control condition, responses to stimuli 7–9 kHz and 16–18 kHz were averaged and served as control deviant. For displaying the results of the initial experiments with different stimulus durations and the control condition, we pooled the posterior electrodes on the left and right hemisphere and the anterior electrodes on the left and right hemisphere. A Wilcoxon Signed Rank Test and subsequent correction of the p-values for multiple comparisons revealed that there was no difference between the potentials recorded from both hemispheres. Obtained p-values were corrected for multiple comparisons using the false discovery rate (FDR, Benjamini and Hochberg [45]). FDR was performed with a MATLAB script from the Mass Univariate ERP Toolbox developed by Groppe et al. [46] adapted to our data.

For latency measurements, we refer to peak latencies (i.e., from the start of the stimulus to the minimum of the respective deflection in a time window of 0 to 50 ms after stimulus onset (N1-peak) and from the start of the stimulus to the maximum of the respective deflection in a time window of 0 to 200 ms after stimulus onset (P2-peak)).

Statistical comparison of evoked potentials from 0 to 250 ms after stimulus onset was done for each data point using a Wilcoxon Signed Rank Test for corresponding sample points (time bin 1 ms). In the time range of 250 to 500 ms no evoked activity was observed, therefore no analysis was performed for this latency range. The resulting p-values were FDR-corrected. For displaying the results, due to a better visibility, the data was downsampled to 0.5 kHz.

The primary data are stored electronically at the Max Planck Institute for Neurological Research, Cologne, in compliance with the data policy of the Max Planck Society. We are happy to make these data available to interested colleagues upon request.

## Results

### Offset Response

**Figure 1:**
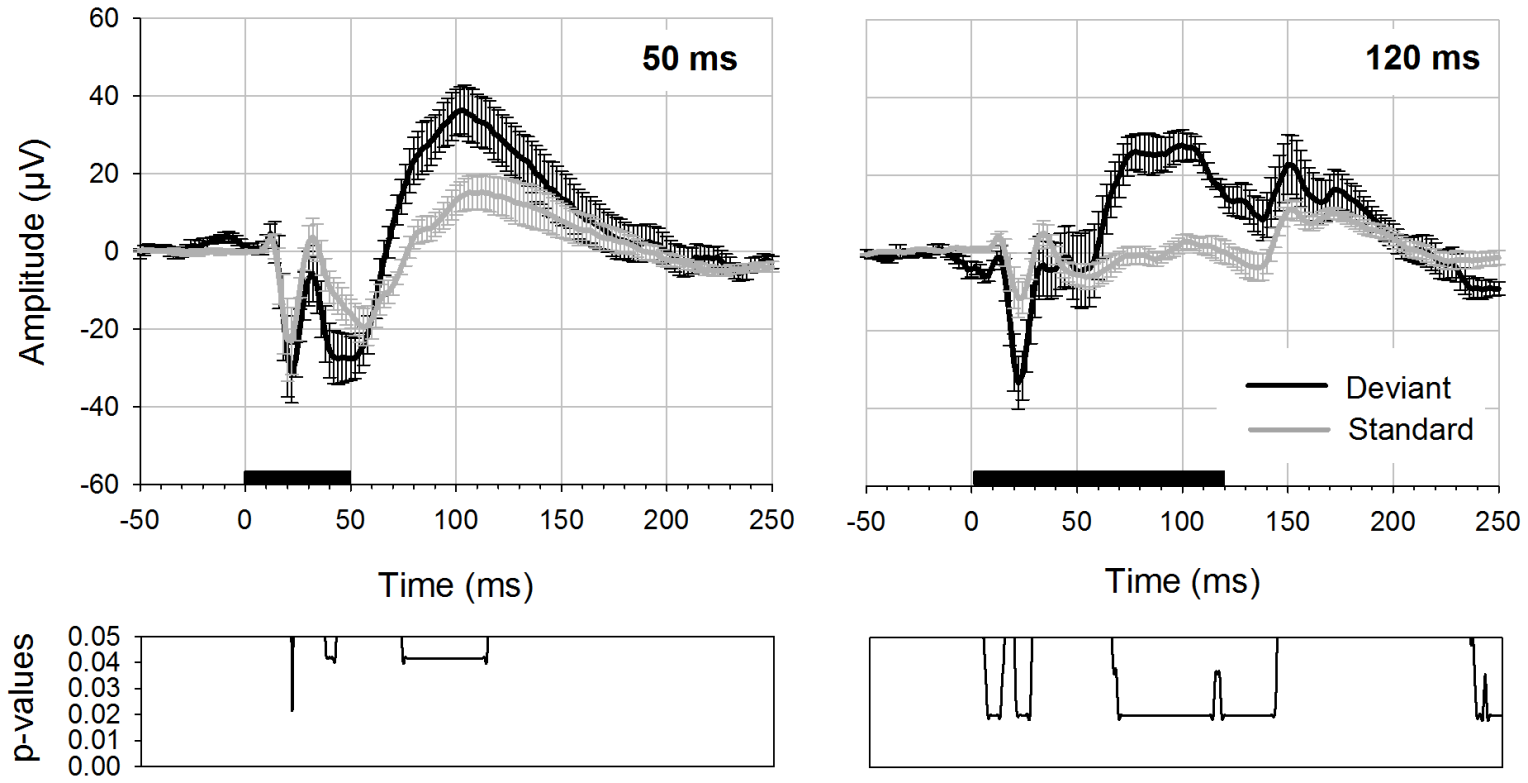
Deviant- and standard-AEPs elicited with stimuli of 50 and 120 ms duration. Deviant (black curve) and standard potential (grey curve) are displayed with errorbars (standard error of the mean). Data is derived from 4 rats. For displaying the results and statistical calculation the data tracks from the anterior recording electrodes were pooled (n = 8). Deviant probability was 0.1. The left figure shows potentials elicited with stimuli of 50 ms duration (40 ms plus 5 ms rise/fall time), the right figure depicts potentials elicited with 120 ms (100 ms plus 10 ms rise/fall time). Black bar on the x-axis shows the stimulus duration. Bottom diagrams indicate p-values for differences between deviant and standard potential (Wilcoxon Signed Rank test, p-values FDR corrected).

In an initial series of experiments with four rats, stimuli of different length were applied in an oddball paradigm. Here, potentials evoked with a stimulus of 50 ms (40 ms plus 5 ms rise and fall time) were compared to potentials evoked with stimuli of 120 ms duration (100 ms plus 10 ms rise and fall time) (Fig. 1). We found stimulus-evoked activity not only at the beginning but also after the end of the stimulus. Notably, this so-called offset response only ceased to overlap with the onset response once stimuli were of 120 ms duration. The experiment resulted in a large difference between standard and deviant potentials regarding the first negative peak (N1) of the onset response and, moreover, lead to another large difference in the latency range of ∼60–130 ms. Significant differences between standard and deviant potential were found in both conditions, and the corresponding latency values, p- and W-values are displayed in Table 1. With a stimulus duration of 120 ms, the late difference between standard and deviant potential fell within the epoch between onset and offset response and had a larger amplitude compared to shorter duration stimuli, therefore we chose this 120 ms stimulus duration (including 10 ms rise and fall time) for all further experiments.

**Table 1:** Significant differences between standard and deviant deflections with respect to stimulus duration.

Stimulus duration (in ms)	Electrode	Latency range (in ms)	W-values	degrees of freedom (df)	p-values
50	A1	22	0	7	p = 0.042
		38–42	0	7	p = 0.042
		74–114	0	7	p = 0.042
120	A1	5–13	1	7	0.02<p<0.036
		19–26	1	7	0.02<p<0.036
		66–143	1	7	0.02<p<0.036
		237–250	1	7	0.02<p<0.036

* p-values are FDR corrected for multiple comparisons.

### Oddball Experiments

**Figure 2:**
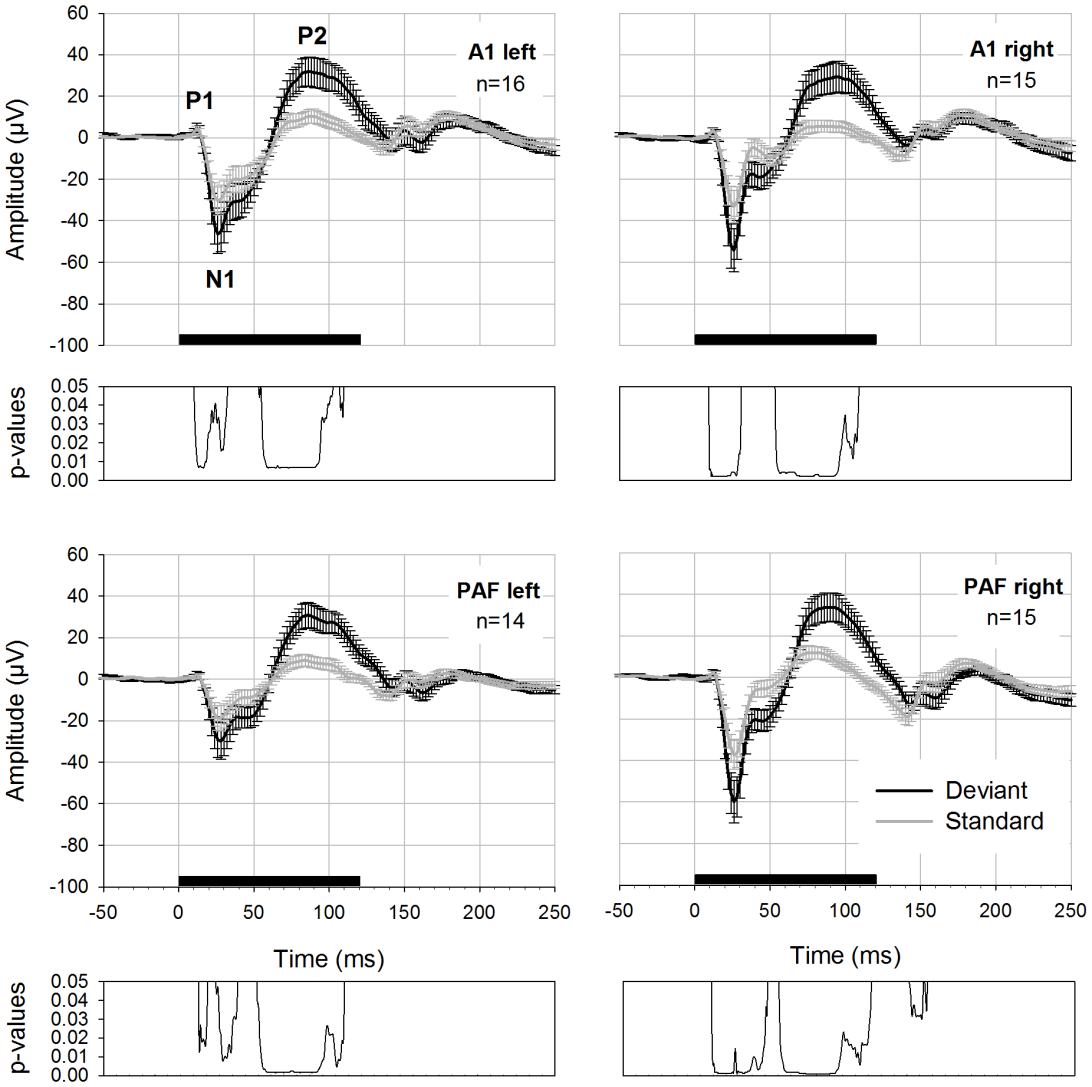
AEPs to deviant and standard stimuli elicited with a deviant probability of 0.2. Deviant (black curve) and standard potential (grey curve) with errorbars (standard error of the mean) displayed for four electrodes. Prominent peaks are labeled (P1, N1, P2). Black bar on the x-axis shows the stimulus duration. Below each graph FDR-corrected p-values are shown.

In the main experiments with 16 rats we tested two different oddball conditions: a high (0.2, 200 deviants +800 standards) and a low deviant probability (0.1, 100 deviants +900 standards). While the anterior left electrode was intact in all animals, the anterior right and posterior right electrodes failed to record evoked activity in one rat, and the posterior left electrode in two rats. These signals were omitted from analysis. Fig. 2 shows the grand average results of the oddball experiment using 0.2 deviant probabilities separately for 4 electrodes. Significant differences between standard and deviant potentials were found in all four electrodes and the corresponding latency values and p- and W-values are reported in Table 2.

**Table 2:** Significant differences between standard and deviant deflections with respect to deviant probability.

Deviant probability	Electrode	Latency range (in ms)	W-values	degrees of freedom (df)	p-values
0.2	A1 left	21–46	4< W <21	15	0.04<p<0.044
		71–128	0< W <22	15	0.007<p<0.049
		133–136	19< W <22	15	0.007<p<0.049
	A1 right	20–44	0< W <15	14	0.002<p<0.024
		71–135	1< W <19	14	0.002<p<0.018
	PAF left	24–30	9< W <13	13	0.016<p<0.031
		38–54	6< W <15	13	0.008<p<0.045
		69–136	0< W <15	13	0.002<p<0.045
	PAF right	19–62	1< W <21	14	0.001<p<0.049
		71–142	0< W <20	14	0.001<p<0.043
		172–185	18< W <21	14	0.032<p<0.049
0.1	A1 left	20–54	1< W <19	15	0.003<p<0.026
		65–138	1< W <22	15	0.003<p<0.04
	A1 right	20–38	0< W <18	14	0.002<p<0.046
		67–129	3< W <18	14	0.002<p<0.046
	PAF left	19–55	1< W <14	13	0.001<p<0.049
		67–133	0< W <16	13	0.001<p<0.049
	PAF right	19–56	1< W <15	14	0.001<p<0.02
		67–140	0< W <20	14	0.001<p<0.048

* p-values are FDR corrected for multiple comparisons.

**Figure 3:**
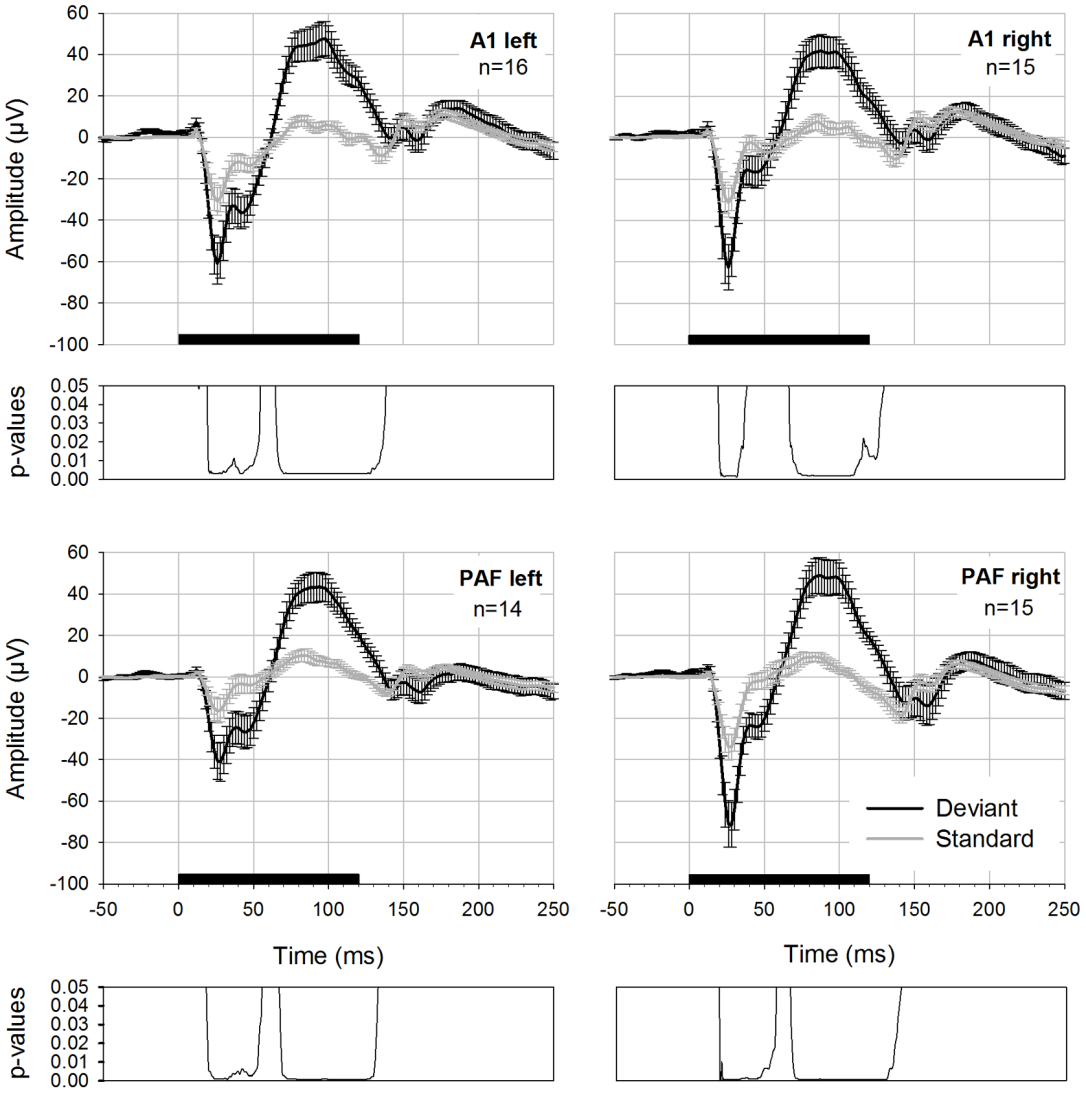
AEPs to deviant and standard stimuli elicited with a deviant probability of 0.1. Deviant (black curve) and standard potential (grey curve) with errorbars (standard error of the mean) displayed for four electrodes. Black bar on the x-axis shows the stimulus duration. Below each graph FDR-corrected p-values are shown.

In Fig. 3 the results of the oddball experiment with 0.1 deviant probabilities are displayed. Significant differences between standard and deviant AEP were found again in all four electrodes. The results of the statistical comparison of both potentials are displayed in Table 2.

The mean latency (± standard error of the mean) for the first negative peak N1 was 29±3 ms (A1 left), 27±0 ms (A1 right), 32±3 ms (PAF left) and 27±2 ms (PAF right) for standard potentials in the 0.1 condition. The latency of the N1 peak in the deviant potential was 26±2 ms (A1 left), 27±1 ms (A1 right), 27±3 ms (PAF left) and 28±0 ms (PAF right). Latencies for the N1 peak in the 0.2 condition in standard potentials were 31±3 ms (A1 left), 28±3 ms (A1 right), 32±3 ms (A1 left) and 28±2 ms (PAF right).

Deviant potentials exhibited the following latencies: 27±2 ms (A1 left), 27±2 ms (A1 right), 31±3 ms (PAF left) and 26±1 ms (PAF right). N1-peaks of deviant potentials tended to exhibit shorter latencies than corresponding standard components and the difference between both latency values reached significance in the 0.2 condition for electrode A1 left (p = 0.041). For the later positive difference in the latency range of P2 it was more difficult to determine a latency value because in averaged standard potentials this deflection was not very pronounced and exhibited a plateau rather than a sharp peak. Mean latencies for P2 in the 0.1 probability condition were 129±15 ms (A1 left), 132±15 ms (A1 right), 103±15 ms (PAF left) and 100±14 ms (PAF right) for standard potentials. For deviant potentials, P2-peaks exhibited the following latencies: 99±11 ms (A1 left), 101±9 ms (A1 right), 85±7 ms (PAF left) and 91±6 ms (PAF right). For the 0.2 probability we found 110±15 ms (A1 left), 126±14 ms (A1 right), 91±14 ms (PAF left) and 95±13 ms (PAF right) for standard potentials. Latencies of deviant potentials were 91±7 ms (A1 left), 109±9 ms (A1 right), 86±7 ms (PAF left) and 98±7 ms (PAF right). Due to the lack of a real peak in standard potentials we did not calculate statistical tests for the positive deflection.

**Figure 4:**
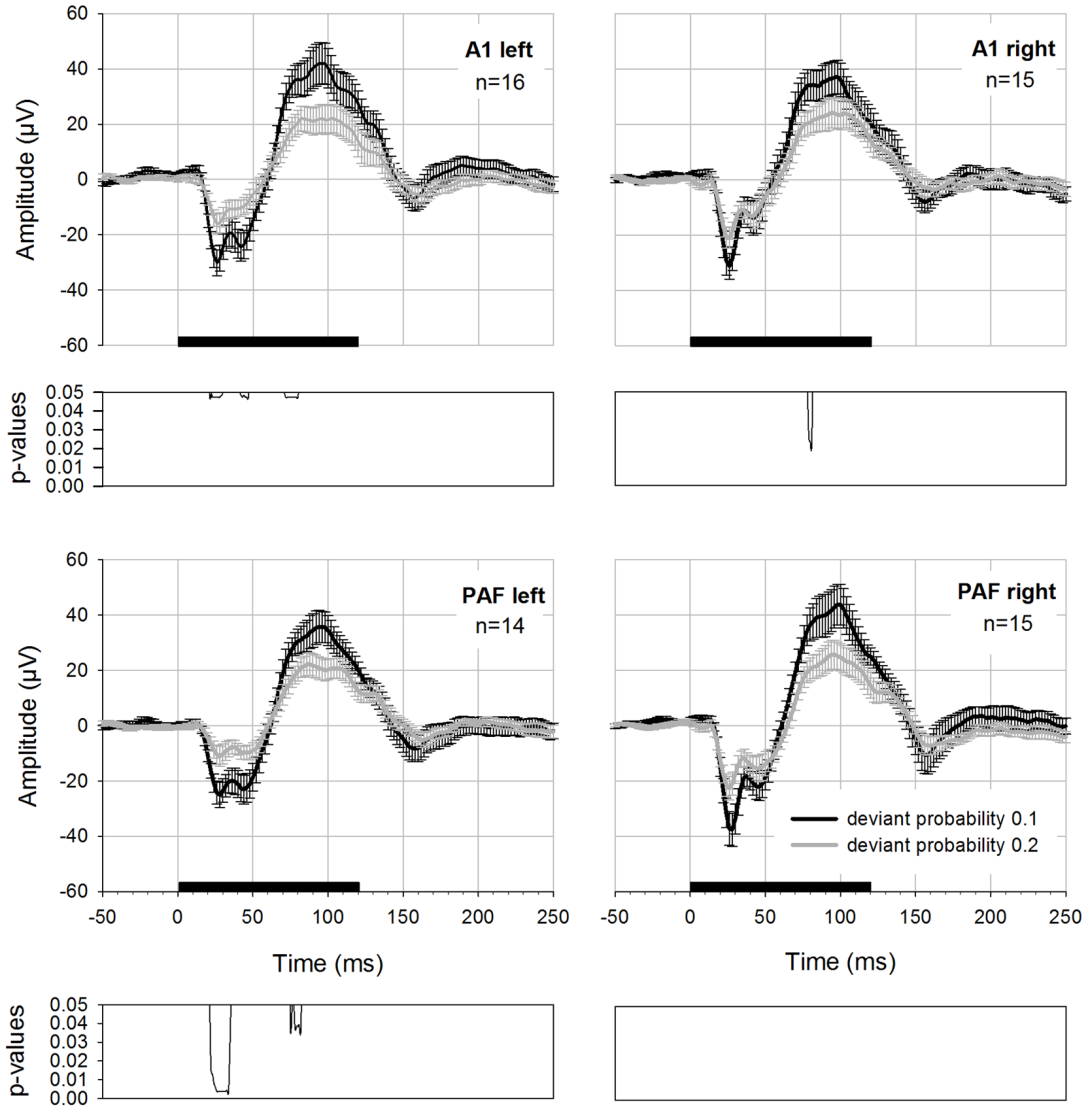
MMN-like activity displayed as difference waveforms for two deviant probabilities. Difference calculated as deviant minus standard potential displayed for four electrodes. Potentials were elicited using an oddball paradigm with deviant probability 0.1 (black curve) and 0.2 (grey curve). Black bar on the x-axis shows the stimulus duration. Below each graph FDR-corrected p-values are shown.

The traditional calculation of mismatch potentials (i.e., difference between deviant and standard AEPs) resulted in an early negative and a late positive component that varied in amplitude depending on the deviant probability used. In Fig. 4 differences for deviant probability 0.1 and 0.2 are compared. Statistical comparison using a Wilcoxon Signed Rank Test (p-values FDR corrected) revealed significantly higher MMN amplitudes for deviant probability 0.1 in three recording electrodes. The statistical differences between both waveforms are reported in Table 3.

**Table 3:** Significant differences between MMN amplitudes of different deviant probabilities (always MMN_0.1_>MMN_0.2_).

Electrode	Latency range (in ms)	W-values	degrees of freedom (df)	p-values
A1 left	22–29	3< W <13	15	0.047<p<0.048
	42–47	13< W <10	15	0.047<p<0.048
	72–80	8< W <12	15	0.047<p<0.047
A1 right	79–81	2< W <3	14	0.023<p<0.025
PAF left	22–35	0< W <4	13	0.004<p<0.038
	75–76	8< W <9	13	0.038<p<0.048
	78–82	7< W <8	13	0.023<p<0.038
PAF right				

* p-values are FDR corrected for multiple comparisons.

In order to evaluate whether there were differences regarding mismatch potentials recorded from primary and secondary auditory cortex, we compared the difference waveforms detected with anterior and posterior electrodes of both hemispheres. There were no statistically significant differences present (corrected p-values >0.05).

### Control Condition

**Figure 5:**
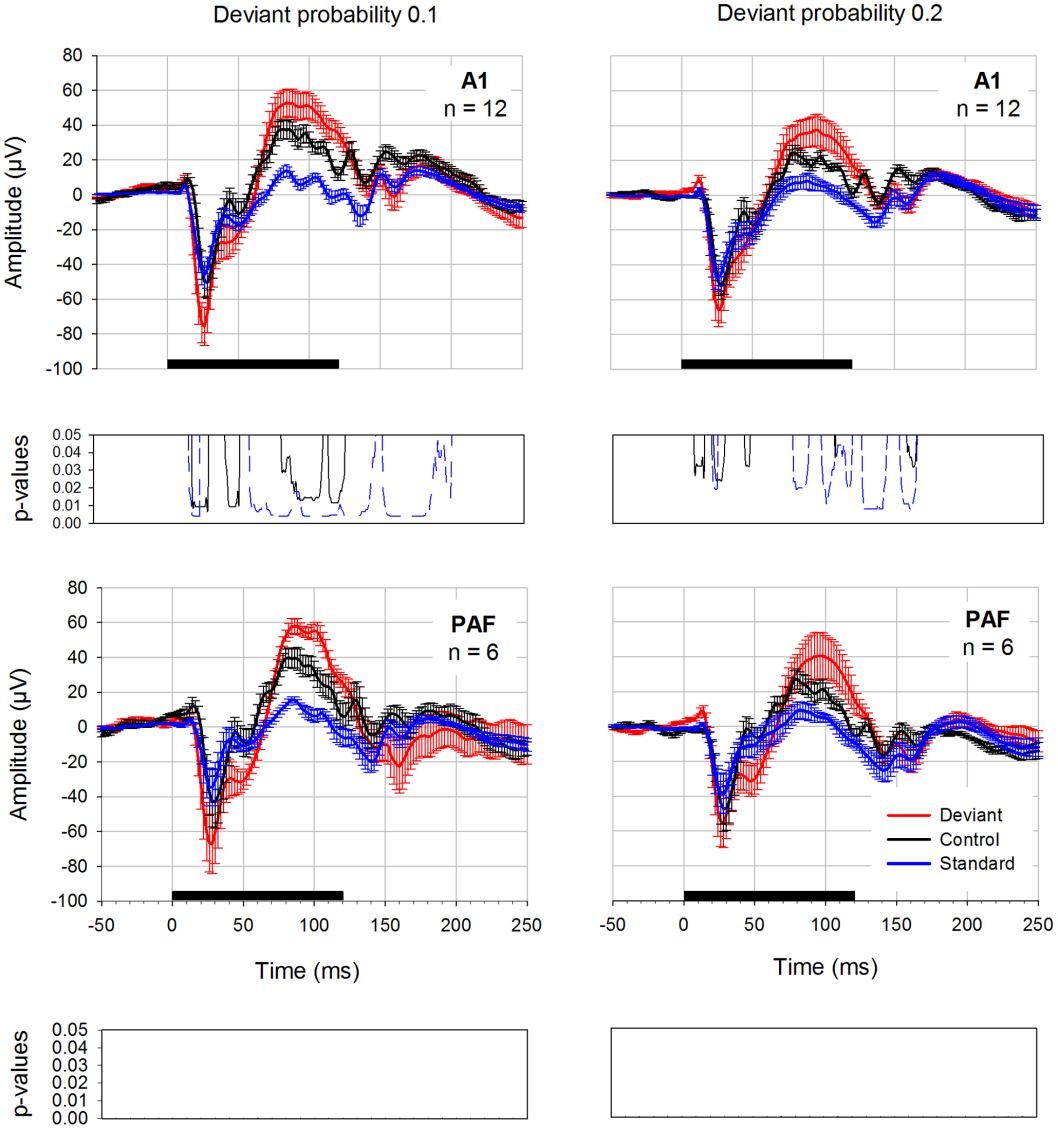
Oddball deviant compared to the equiprobable control condition. Oddball deviant (red curve), control “deviant” (black curve) and oddball standard (blue curve) potentials were elicited with either 0.1 deviant probability (diagrams on the left side) or 0.2 deviant probability (diagrams on the right side). Data is derived from 6 rats. The posterior electrodes on the left and right hemisphere as well as anterior electrodes on the left and right hemisphere were pooled for displaying the results and statistical calculation. Black bar on the x-axis shows stimulus duration. Below each graph FDR-corrected p-values are shown. The black curve displays the differences between oddball deviants and control deviants whereas the blue curve displays significant differences between oddball standard and control deviant.

In order to differentiate between adaptation and deviance detection mechanisms we applied a control condition suggested by Jacobsen and Schröger [39] in 6 rats. This used both of the two deviant probabilities from the oddball experiments, resulting in two controls (equiprobable control 0.1, equiprobable control 0.2). “Deviants” had the same frequencies and rate of presentation as the deviants used in the oddball condition, but were presented among several other stimuli with the same rate instead of a homogeneous sequence of standards. The results of these measurements are presented in Fig. 5. The data was pooled for the anterior electrodes (left and right hemisphere) and for the posterior electrodes (left and right hemisphere). In addition, deviant and standard potentials elicited with the classical oddball paradigm are displayed in this figure. Deviant potentials in the oddball condition had higher amplitudes compared to “deviants” in the equiprobable control condition. For probability 0.1, differences between deviants recorded in the oddball condition and “deviants” in the equiprobable control condition were found. In addition, there were significant differences between oddball standard and control “deviant” potentials (Table 4). These differences were limited to the anterior electrodes; in the posterior electrodes p-values did not reach significance after FDR correction. For probability 0.2, again, oddball deviant and standard were significantly different from control “deviant” in the anterior electrodes (Table 4). There were no significant differences after correction of the p-values for multiple comparisons in the posterior electrodes.

**Table 4:** Significant differences between the deviant in the control condition and the deviant in the oddball condition and differences between control deviant and the standards in the oddball condition.

Deviantprobability		Electrode	Latency range (in ms)	w-values	degrees offreedom	p-values
0.1	Control vs MMN_deviant	A1	19–33	0< W <4	11	0.009<p<0.018
			42–51	0< W <7	11	0.009<p<0.037
			81–110	2< W <8	11	0.013<p<0.048
			114–125	1< W <6	11	0.012<p<0.03
		PAF				
	Control vs Standard_MMN	A1	17–24	0< W <7	11	0.004<p<0.018
			59–146	0< W <10	11	0.004<p<0.037
			152–199	0< W <11	11	0.004<p<0.047
		PAF				
0.2	Control vs MMN_deviant	A1	7–14	2< W <5	11	0.032<p<0.044
			21–27	0< W <3	11	0.024<p<0.032
			42–45	3< W <5	11	0.032<p<0.044
			105	W = 4	11	p = 0.037
			155–161	3< W <5	11	0.032<p<0.044
		PAF				
	Control vs MMN_standard	A1	19–23	3< W <8	11	0.019<p<0.044
			76–86	3< W <6	11	0.019<p<0.031
			96–117	1< W <8	11	0.011<p<0.044
			124–141	0< W <8	11	0.008<p<0.044
			150–162	0< W <8	11	0.008<p<0.044
		PAF				

* p-values are FDR corrected for multiple comparisons.

## Discussion

In this study, we recorded MMN under natural conditions from primary auditory cortex and posterior auditory field in awake and unrestrained rats. These recordings were performed in both hemispheres simultaneously, using a wireless recording system. Applying a classical oddball paradigm with optimized spectral stimulus properties (bandpass filtered noise stimuli adapted to the rats’ optimal hearing range) and stimulus duration, we found robust differences between deviant and standard AEP at all four electrodes. These MMN-like responses shared some key morphological and functional characteristics with human MMN; for example, their amplitudes increased with decreasing deviant probability. For the first time, we provide a replication of this effect across four electrodes, i.e., in primary and secondary auditory areas of both hemispheres. Furthermore, when removing the predictive context in a control (equiprobable) condition, the amplitudes of these responses were significantly reduced in primary auditory cortex.

### Morphology of Potentials

In an initial study with four rats we used acoustic stimuli of different length to investigate the impact of this change on cortical evoked potentials. Obligatory components of each potential were an early fast negative deflection and a late slower positive deflection, which concurs with earlier studies in rats [47], [48]. Moreover, the latencies of prominent peaks are in accordance with previously presented results [47]. Onset potential components were followed by offset responses that consisted again of a negative-positive deflection but of smaller amplitude. This was also found in a study recording local field potentials in rat’s auditory cortex [30].

Most previous MMN-studies in rats have used shorter stimuli than in this study [31], [33]–[35], [40]. This may be an important difference as we found that for stimuli shorter than 100 ms MMN-responses might have overlapped with the offset response. In contrast, with stimuli of 120 ms duration, the maximum difference between standard and deviant potential was located between the on- and the offset response. For our main experiments we therefore chose a stimulus duration of 120 ms.

### Comparison with Previous Studies

When comparing our results to previous MMN studies in rats, it is important to take into account differences in the physical attributes (carrier frequency and duration) of the acoustic stimuli used and differences in control conditions. In this study, we tried to improve previous experimental protocols by optimizing the auditory stimulus properties: in initial tests, we carefully chose the most suitable frequencies (adapted to the rats’ hearing range) and stimulus durations for eliciting MMN.

In the main experiments with 16 rats we tested two different oddball conditions: a high (0.2) and a low (0.1) deviant probability. In both conditions deviant potentials had larger amplitudes than standard potentials. The calculated difference waveforms (averaged evoked deviant potential minus the averaged evoked standard potential) resulted in a large amplitude biphasic wave. The early negative as well as the late positive potential component increased with decreasing deviant probability. Notably, this result mirrors the findings from the human MMN literature [49]–[54].

We found no striking latency or shape differences, i.e., additional potential components, between the four electrodes. In guinea pigs [23], cats [41] and humans [55] additional potential components in response to oddball deviants have been found and interpreted as MMN. These potential components seem to be generated also in secondary auditory cortex. In our study, we did not find any differences between mismatch responses recorded from primary and secondary auditory fields.

### The Effect of Anesthesia on MMN

The most important factor to consider when comparing rat MMN studies, however, may be the effect of anesthesia. One key goal of our study was to obtain MMN recordings from awake, non-anaesthetized animals, while most previous MMN studies in rats were conducted under anesthesia. Fentanyl-medetomidine anesthesia, for example, was shown to change the shape of AEPs and reverse the polarity of MMN-like potentials in rats [28]. Under urethane anesthesia, AEPs exhibited a slow positive component followed by a smaller slow negative component [40] while fast onset responses were completely absent. However, mismatch responses of positive polarity were reported 60 to 100 ms after stimulus onset for melodically ascending deviants. Under pentobarbital-sodium anesthesia, on the contrary, fast onset responses are preserved [33], [34]. The synaptic effects of these (and most other) anesthetic agents are not fully understood and, as shown by the above examples, can significantly impact on MMN responses. By using awake animals, our protocol eschews potentially confounding interactions between the physiological mechanisms underlying MMN and anesthesia effects. This is of particular importance when establishing rodent MMN models as a platform for studying pathophysiological mechanisms in diseases linked to reduced MMN expression, such as schizophrenia.

To our knowledge, there is only one other study recording AEPs to oddball stimulation epidurally in awake rats [28]. The potentials shown are similar to our results regarding the first two peaks. AEPs exhibited an initial small positive response followed by a negative peak (named “N29” by the authors to indicate its latency). This peak corresponds to our N1 peak that was detected with a similar latency. Furthermore, the positive peak P38 reported by Nakamura et al. appears to correspond to the positive deflection around 40 ms in our study. However, a more prominent finding in our study was the large amplitude positive peak (P2) that commenced around 55 ms, reached its maximum amplitude around 100 ms and lasted until the end of the stimulus. This difference might be explained by the frequency of the acoustic stimuli used in the study of Nakamura et al. [28] that were located at the lower end of the rats' hearing range (2500 and 3600 Hz). The stimuli applied in our study fit the rats hearing ability better and consequently evoked higher amplitude AEPs with more pronounced peaks and overall longer lasting sustained activity. Nevertheless, the difference wave comparing the higher frequency deviants (3600 Hz) with higher frequency standards presented by Nakamura et al. [28] resembles the difference waveforms found in our study. It starts with a double negative peak followed by a waveform of positive polarity.

### Relationship between MMN and SSA

Both SSA and prediction error signals are potential mechanisms of MMN. In the equiprobable control condition, a prediction cannot be established because there is no recurrent standard stimulus. Furthermore, the control “deviant” was presented with the same average rate and the same overall stimulation duty cycle as the deviant in the oddball condition. Hence, the amplitude differences we found between the standard response in the oddball condition and the “deviant” response in the equiprobable control condition are most likely caused by SSA, reducing the amplitude of the standard potential. On the other hand, amplitude differences which occurred between the deviant in the oddball condition and the “deviant” in the equiprobable condition, particularly those of the P2 deflection (see above), could be interpreted as resulting from prediction errors. However, it is possible that cross-frequency adaptation due to the relatively close spacing of our frequencies [56] may have contributed to an overall amplitude reduction in the control condition (cf. Taaseh et al. [56]). Under our experimental design, it cannot be determined how extensive this possible contribution of cross-frequency adaptation was altogether. While this is clearly a limitation of the present study, some useful information on this potential constraint can be gathered from previous studies. In a recent study using LFP and multiunit recordings (Farley et al., 2010), stimulus frequencies in the equiprobable control were equally and sufficiently broadly spaced so that cross-frequency adaptation did not occur. For fast responses (latency 20 ms) SSA was the only detectable mechanism, whereas for late responses in the same latency range as in our study (around 110 ms) there was no evidence for either SSA or prediction error because of high response variability. Other studies (Nakamura et al., 2011; von der Behrens et al., 2009) showed that adaptation indeed plays a role for the late positive wave, but in a less pronounced fashion as for the first negative peak. Taken together, these studies show that contribution of SSA to MMN seems reliable, while not providing a stringent demonstration for the existence of prediction error response in rodent. On the contrary, in humans, the evidence for involvement of prediction error processing in MMN is much stronger. As summarized by Näätänen et al. [57] and Garrido et al. [3], some properties of the human MMN cannot be explained by adaptation and contemporary MMN theories have already begun to integrate these accounts within a unified explanation of MMN. In this predictive coding framework [3] prediction error dependent synaptic plasticity of inter-regional connections implements the online adjustment of a predictive model, while, at faster timescales, adaptation tunes the relative postsynaptic sensitivity to top-down predictions and bottom-up stimulus information. We will examine this suggestion in future studies.

The presented study indicates that robust MMN-like responses can be obtained in awake and unrestrained rats. This provides a basis for future experimental investigations of the mechanisms that underlie MMN generation without having to worry about the potential confounds of anesthesia. Establishing anesthesia-independent settings for probing rodent analogues to the human MMN are important for facilitating the detection of therapeutic targets at the cellular level. Knowledge of these targets is likely to help guiding the development of drugs for treating the disorders that have been shown to be accompanied with reduced MMN responses, such as schizophrenia.
